# Optimization of selective stimulation parameters for multi-contact electrodes

**DOI:** 10.1186/1743-0003-10-25

**Published:** 2013-02-27

**Authors:** Lee E Fisher, Dustin J Tyler, Ronald J Triolo

**Affiliations:** 1 Case Western Reserve University, Cleveland, OH 44106, USA; 2 Louis Stokes Cleveland Department of Veterans Affairs Medical Center, Cleveland, OH 44106, USA

**Keywords:** Functional neuromuscular stimulation, Selectivity, Multi-contact electrodes

## Abstract

**Background:**

Multi-contact stimulating electrodes are gaining acceptance as a means for interfacing with the peripheral nervous system. These electrodes can potentially activate many independent populations of motor units within a single peripheral nerve, but quantifying their recruitment properties and the overlap in stimulation between contacts is difficult and time consuming. Further, current methods for quantifying overlap between contacts are ambiguous and can lead to suboptimal selective stimulation parameters. This study describes a novel method for optimizing stimulation parameters for multi-contact peripheral stimulating electrodes to produce strong, selective muscle contractions. The method is tested with four-contact spiral nerve-cuff electrodes implanted on bilateral femoral nerves of two individuals with spinal cord injury, but it is designed to be extendable to other electrode technologies with higher densities of contacts.

**Methods:**

To optimize selective stimulation parameters for multi-contact electrodes, first, recruitment and overlap are characterized for all contacts within an electrode. Recruitment is measured with the twitch response to single stimulus pulses, and overlap between pairs of contacts is quantified by the deviation in their combined response from linear addition of individual responses. Simple mathematical models are fit to recruitment and overlap data, and a cost function is defined to maximize recruitment and minimize overlap between all contacts.

**Results:**

Results are presented for four-contact nerve-cuff electrodes stimulating bilateral femoral nerves of two human subjects with spinal cord injury. Knee extension moments between 11.6 and 43.2 Nm were achieved with selective stimulation through multiple contacts of each nerve-cuff with less than 10% overlap between pairs of contacts. The overlap in stimulation measured in response to selective stimulation parameters was stable at multiple repeated time points after implantation.

**Conclusions:**

These results suggest that the method described here can provide an automated means of determining stimulus parameters to achieve strong muscle contractions via selective stimulation through multi-contact peripheral nerve electrodes.

## Background

Multi-contact stimulating electrodes have been gaining acceptance as a means for interfacing with peripheral nerves in functional neuromuscular stimulation (FNS) systems [1-3]. These electrodes allow for a high density of contacts to be placed around or in peripheral nerves to independently activate multiple fascicles and motor units.

Independent activation can allow for the control of multiple functions with a single electrode and for recruitment of multiple populations of agonist motor units within a single muscle [4,5]. Control of multiple functions with a single electrode could reduce the number of implantation sites required to produce a variety of functional joint moments for FNS systems [5]. Control of multiple agonist motor unit populations with a single electrode could allow for better control of joint moment by varying the number of motor units recruited by the electrode [4]. Further, by alternating stimulation of multiple agonist motor unit populations, it may be possible to reduce stimulation duty cycle and prolong the time of muscle contractions before the onset of fatigue [6]. To take advantage of these benefits of multi-contact electrodes, it is crucial to be able to measure and minimize overlap in recruitment between the contacts within these electrodes. Overlap in recruitment may limit the options available to control the response to stimulation. Repeated activation of the same motor units by multiple contacts may also contribute to rapid fatigue.

Determining selective stimulation parameters is a complex problem when multiple contacts activate agonist populations of motor units. It is difficult or impossible to separate populations of agonist motor units based on their lines of action or resultant joint moments. Ideally, if the spatial relationship between the electrode and the fascicles within the nerve was known *a priori*, it would be possible to select stimulation parameters based on computational models and estimates of activation of the neural tissue. Peripheral nerves, however, have a highly branched structure, and there is a high degree of person-to-person variability in the anatomy of those nerves, both in branching structure and in the location of fascicles that innervate specific muscles [7,8]. Because current state-of-the-art imaging technology is insufficient to accurately visualize the fascicular structure of nerves *in vivo*, it is not possible to know what the spatial relationship is between the contacts within a nerve-cuff electrode and the fascicles and respective muscles the electrode will activate. Stimulation overlap must, therefore, be inferred from indirect measurements [9]. Electromyogram recordings are commonly used to infer selectivity, but the strong dependence on the spatial relationship between the recording electrodes and the activated motor units limits the practical utility of this method and can lead to over- or underestimation of selectivity [4,10].

Another method for quantifying selectivity takes advantage of the concept that motor units in their absolute refractory period, which lasts between 1.5 and 2.1 ms, will not respond to stimulation [1,3,6,11-15]. Therefore, if a stimulus pulse is applied through one electrode contact within 2.1 ms after a pulse is applied through another electrode contact, motor units activated by the first stimulus will not respond to the second stimulus. This means that, if there is overlap in the stimulation fields of two contacts, the resultant force generated when one contact is activated within 2.1 ms after the other will be less than the linear sum of the individual forces when each contact is activated separately. Conversely, the forces generated by stimulating two completely independent populations of motor units will add linearly, even if one population is stimulated while neurons from the other population are in their absolute refractory period [14]. By varying stimulation parameters including pulse amplitude and pulse width, it may be possible to minimize the deviation from linear addition, and thereby minimize stimulation overlap, while maximizing the magnitude of the force generated by each independent motor unit population to selectively produce strong muscle contractions to lock the knees and improve FNS-assisted standing.

While this method provides a useful way to quantify overlap between two contacts, it does not easily scale to larger numbers of contacts. To create a metric of overall overlap for an entire multi-contact electrode, others have used this method to quantify overlap between pairs of contacts within the electrode, and then averaged all of those overlaps [3,6]. While this method provides some insight into the general amount of overlap for a multi-contact electrode, it does not provide a clear means of tuning stimulation parameters for each individual contact to reduce overlap while generating functionally useful stimulated joint moments. Furthermore, as the number of contacts within the electrode increases, the number of pairwise combinations of contacts that must be considered for this method increases exponentially. This can quickly lead to impossibly large data sets as the number of contacts increases.

In this report, we propose a method for choosing optimal selective stimulation parameters for multi-contact electrodes that minimizes overlap between adjacent contacts while maximizing the joint moment produced by stimulating through each contact. We rely on the method described above to quantify overlap between pairs of contacts, with the addition of a set of mathematical models to reduce the data requirements for characterizing the electrodes, and a cost function that acts to minimize all pairwise overlaps while simultaneously maximizing all joint moments. We show that this method can efficiently characterize overlap and selectivity for multi-contact electrodes to produce strong muscle contractions with little or no overlap between stimulated motor unit populations. While the method is designed to accommodate electrodes with high densities of contacts, we test it clinically with the four-contact spiral nerve-cuff electrode developed at Case Western Reserve University (CWRU). We demonstrate that the method can select stimulation parameters that generate strong contractions with low overlap between contacts in this electrode, and that the selective responses are stable over months after implantation.

## Methods

The process we propose to quantify and optimize selective stimulation for multi-contact electrodes consists of four fundamental steps. First, the response to stimulation through the multi-contact electrode and the overlap between pairs of contacts are quantified. These responses are the forces generated by muscle twitches, elicited by single stimulus pulses. Muscle twitches are less likely to cause fatigue than tetanic contractions and can be collected more quickly. Next, the relationship between twitch and tetanic responses to stimulation is quantified. This relationship provides a scaling factor so that the twitch responses can be converted to more functionally relevant tetanic responses. Third, mathematical models are fit to the scaled recruitment and overlap data. These models serve the dual purposes of reducing the size of the data set required for optimization and providing a mathematical framework over which optimization can be performed. Finally, the scaled recruitment and overlap models are used as inputs to a cost function that can be minimized to provide optimal selective stimulation parameters.

### Subject selection and multi-contact electrodes

**Figure 1:**
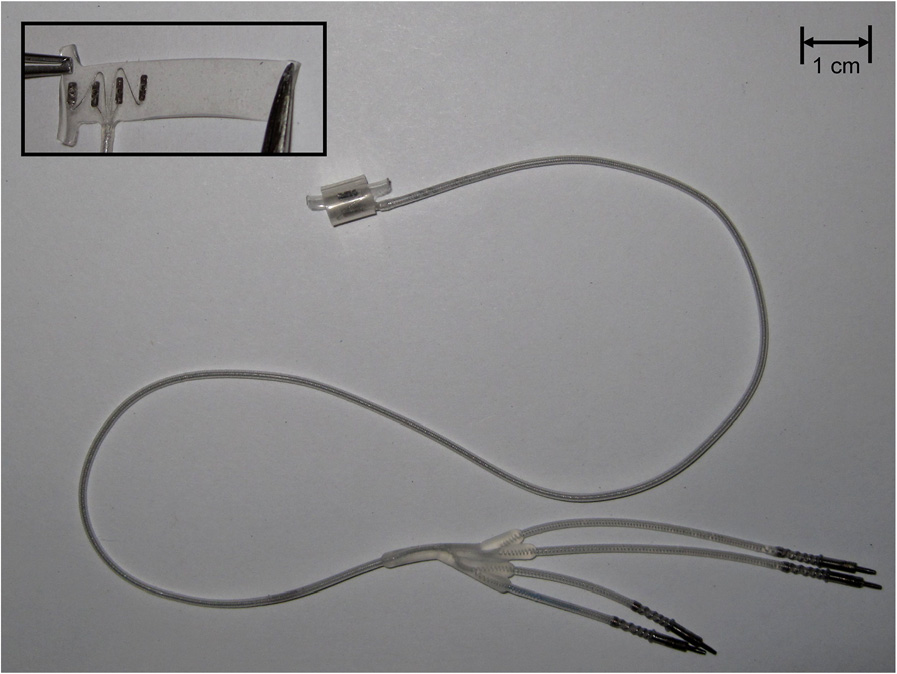
**The CWRU spiral nerve-cuff electrode.** The CWRU four-contact spiral nerve-cuff electrode was implanted around bilateral femoral nerves to stimulate the knee extensor muscles. Each contact is connected to an independent channel of stimulation.

The CWRU self-sizing four-contact spiral nerve-cuff electrode (Figure 1) was used in development and testing of this method for optimizing selective stimulation parameters [16-18]. A total of four nerve-cuffs were implanted chronically around bilateral femoral nerves to stimulate the knee extensors of two volunteers with motor-complete spinal cord injury (Subject 1: level C7, ASIA B and Subject 2: level T11, ASIA B). The nerve-cuffs, which have four contacts that can be controlled independently, were sized so that any two adjacent contacts were separated by 90° around the circumference of the nerve. All contacts were connected to independent channels of an implanted stimulator capable of generating monopolar, charge-balanced biphasic stimulus pulses [19,20]. All stimuli had current amplitudes of 1.4 mA for Subject 1 and 0.8 mA for Subject 2. These values were selected as they provided the largest range between the threshold and maximal responses to stimulation of the available amplitudes from the implanted stimulator. Initial testing of nerve-cuff selectivity was performed at 15 and 14 weeks post-implantation for Subjects 1 and 2, respectively, with additional stability testing performed up to 53 and 37 weeks post-implantation.

Informed consent was acquired prior to participation in any experiments, and all experimental protocols were approved by the Institutional Review Board of MetroHealth Medical Center, Cleveland, OH.

### Recruitment and overlap characterization

For the first step in optimizing selective stimulation parameters, the response to stimulation and the overlap between pairs of contacts were characterized. With the knee fixed at 20° of flexion and one axis of a 6 degree-of-freedom load cell (JR3, Woodland, CA) aligned with the knee joint center, isometric knee extension moment was recorded in response to stimulus pulses applied to the femoral nerve through each contact of the cuff electrodes. Data were low-pass filtered at 31.25 Hz and sampled at 150 Hz.

To characterize the response to stimulation, pulse width modulated recruitment curves were collected. To characterize overlap between pairs of contacts, a stimulus pulse was applied through one contact, followed by a 2 ms time delay, and then a pulse through a second contact. The pulse widths of all stimuli were varied between 1 and 255 us.

For some multi-contact electrodes, it is possible to reduce the size of the data set by eliminating pairwise combinations of contacts that are not adjacent to each other, since elimination of overlap between adjacent contacts will also eliminate overlap between non-adjacent contacts. For example, in the case of an eight contact Flat Interface Nerve Electrode, this would reduce the number of possible pairwise combinations from 28 to 16 [21,22]. In the case of the four-contact nerve-cuff, all contacts are adjacent to each other, so all six pairwise combinations were considered in this study.

### Twitch/tetanic relationship

While the twitch response to stimulation can be collected quickly and with less likelihood of causing fatigue, the tetanic response to stimulation is functionally relevant. Studies in both animals and humans have demonstrated that there is a linear relationship between the shape of isometric twitch and tetanic recruitment curves, characterized by a single scalar multiplier [4,23]. To quantify this scaling factor, twitch and tetanic responses to stimulation were recorded with the knee held in 20° of flexion. The ratio of the maximum twitch and tetanic responses was used as a scaling factor.

### Mathematical models of recruitment and overlap

Fitting mathematical models to recruitment and overlap data reduces the size of the data set required for characterizing the electrodes while also providing a framework for optimization of stimulation parameters. To determine the best form of functions for recruitment and overlap, mathematical functions were fit to knee extension moment data in response to stimulation through individual contacts or pairs of contacts within each electrode. For each contact or pair of contacts, 48 data points were collected, with subsets of 32 recruitment or overlap data points fitted to a variety of models and separate subsets of 16 data points used to test for goodness-of-fit (GOF). For recruitment data, 1^st^ through 5^th^ order polynomial, sigmoid, Gaussian, and Gompertz functions were tested. For overlap data, which are two-dimensional since pulse width can be controlled independently for both contacts in a pairwise combination, 1^st^ through 5^th^ order two-dimensional polynomials were tested. To determine GOF, coefficients of determination (R^2^), and the corrected Akaike Information Criterion (AICc) were calculated for each model. AICc is a measure of how well a model fits a set of data relative to the number of parameters in that model [24]. Models that achieved the best fits were selected for implementation in the optimization described in the following section.

### Optimization of selective stimulation parameters

Achieving selectivity of stimulation necessarily creates a trade-off between large stimulus levels with large joint moments and small stimulus levels with low overlap. It is, therefore, useful to treat selectivity as an optimization problem, where the goal is to choose the best stimulation parameters to maximize joint moment while minimizing overlap, using a cost function of the form

$$C\left(\bar{\mathrm{PW}}\right)=-\omega_{0}M_{T}\left(\bar{\mathrm{PW}}\right)+\omega_{1}O_{T}\left(\bar{\mathrm{PW}}\right)$$

where *PW* is an *N*-dimensional vector of pulse widths of stimulus pulses for an *N*-contact electrode, *O*_*T*_ quantifies the overlap of all contacts within the electrode, *M*_*T*_ quantifies the joint moment generated by all contacts within the electrode, and *ω*_*0*_ and *ω*_*1*_ are weighting factors. By minimizing this cost function, *M*_*T*_ will be maximized and *O*_*T*_ will be minimized, producing the strongest possible contractions with the least possible overlap in stimulation between contacts.

The joint moment term, *M*_*T*_, is defined here as

$$M_{T}\left(\bar{\mathrm{PW}}\right)=\sum_{i=1:N}M_{i}\left(PW_{i}\right)/\sum_{i=1:N}\max\left(M_{i}\right)$$

where *M*_*i*_ is the moment generated when stimulating through contact *i*, which is described mathematically by the model function previously fit to recruitment data. The sum of these functions is divided by the sum of the maxima of the functions to normalize the joint moment term. Because of this normalization, the overall joint moment term ranges between 0 and 1, while the summation of individual moments allows stronger joint moments to be weighted more heavily than weaker moments.

Overlap for a pair of contacts is quantified by the deviation from linear addition when stimulation is applied through one contact shortly after stimulation through another contact. As long as the time delay between stimulus pulses is less than the refractory period of the stimulated motor axons and joint angle remains constant, the resultant joint moment will be the difference between linear addition of the moments when each contact is stimulated individually and the response due to the overlap in stimulation between the two contacts. This can be expressed as

$$M_{i\cup j}\left(PW_{i},PW_{j}\right)=M_{i}\left(PW_{i}\right)+M_{j}\left(PW_{j}\right)-M_{i\cap j}\left(PW_{i},PW_{j}\right)$$

where *M*_*i*U*j*_ is the moment generated when stimulating through two contacts with a short time delay, *M*_*i∩j*_ is the overlap between contacts *i* and *j*, and *M*_*i*_ and *M*_*j*_ are the moments generated when stimulating through contacts *i* and *j*, respectively. By rearranging these terms, the overlap in stimulation can be calculated for a pair of contacts within an electrode.

To take all of these pairwise overlaps into account, while normalizing the overlap so that its weighting is controlled relative to *M*_*T*_, *O*_*T*_ is defined as

$$O_{T}\left(\bar{\mathrm{PW}}\right)=\frac{2}{N^{2}-N}\sum_{i=1:N-1}\sum_{j=2:N}\frac{M_{i\cap j}\left(PW_{i},PW_{j}\right)}{M_{i\cup j}\left(PW_{i},PW_{j}\right)}$$

which ranges between 0 when there is no overlap and 1 when there is 100% overlap. Note also that in this equation, *i≠j*. During this study, a 2 second interpulse interval was used when stimulus pulses were applied to a pair of contacts within an electrode.

Since both *O*_*T*_ and *M*_*T*_ are normalized, the weighting factors *ω*_*0*_ and *ω*_*1*_ can be used to emphasize larger joint moments or lower overlap, depending on the particular application. For this study, the terms were weighted equally, i.e., *ω*_*0*_ = *ω*_*1*_.

To ensure that either sufficiently large joint moments or sufficiently small overlaps are achieved, a linearly increasing penalty was added to the cost function if joint moment for any contact was less than 5 Nm or overlap was greater than 10% between any two contacts. The slope of these penalties can be tuned to tighten or relax the constraints on minimum joint moment or maximum overlap.

A direct search optimization algorithm (Matlab, Natick, MA) was used to find the minimum of the cost function and the optimal set of pulse widths for selective stimulation.

### Stability of overlap

To achieve reliable control in an FNS system, it is important that the response to stimulation is stable over time. Selectivity is a function of both muscle strength and stimulation overlap. While it is expected that muscle strength will change over time as the user exercises and builds muscle mass, if the interface between the electrode and nerve is stable, overlap is less likely to change over time. To test the stability of stimulation, the amount of overlap between pairs of contacts was quantified at multiple time points after implantation. At the first time point, optimal stimulation parameters were chosen by the algorithm described above, and at each subsequent time point, overlap in stimulation between all pairs of contacts was measured using those same stimulation parameters. A one-tailed Student's *t*-test was used to determine if the mean overlap for any pair of contacts was greater than 10%.

## Results

### Electrode characterization and mathematical models

**Figure 2:**
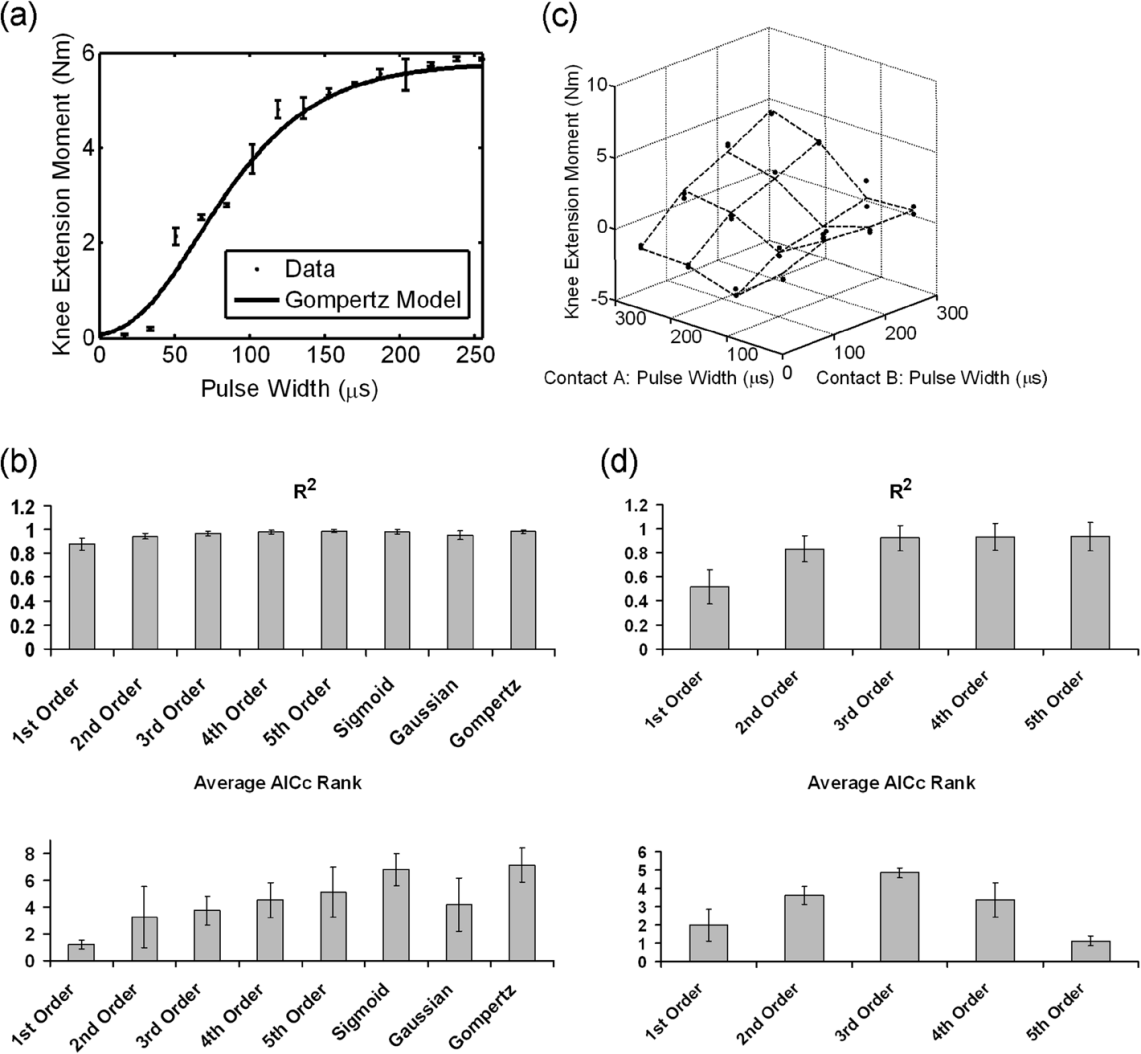
**Recruitment and overlap data, and goodness-of-fit of mathematical models.** (**a**) Recruitment data for a single contact, fit with a Gompertz model. (**b**) Average R^2^ and AICc ranking of eight models fit to recruitment data. A higher AICc rank score denotes a better fit. (**c**) Overlap data for two contacts within a nerve-cuff, fit with a third-order polynomial model. (**d**) Average R^2^ and AICc ranking of five models fit to overlap data.

Shown in Figure 2a are the responses to stimulation through one contact of a nerve-cuff electrode (dots) and a Gompertz model fit to those data (line). Figure 2b shows the average R^2^ and AICc calculated for all eight contacts within the four nerve-cuff electrodes. Since AICc is a ranking of the appropriateness of each of the models, it ranges from 1 to 8, with 1 being the worst model and 8 being the best. From these results, the Gompertz function, which has the form

$$M_{i}\left(PW_{i}\right)=a_{2}e^{a_{1}e^{a_{0PW_{i}}}}$$

achieves the best GOF (R^2^ = 0.98 ± 0.01) and is the most appropriate model of the response to stimulation.

Shown in Figure 2c is an example of overlap data between two contacts within one of the nerve-cuff electrodes (dots) as well as an example of a third-order polynomial model fit to those data (dashed lines). Figure 2d shows the average R^2^ and AICc calculated for all pairwise combinations of contacts for all four nerve-cuff electrodes. From these results, the third-order polynomial, which has the form

$$\begin{array}{l} M_{i\cup j}\left(PW_{i},PW_{j}\right)=a_{9}PW_{i}{}^{3}+a_{8}PW_{j}{}^{3}+a_{7}PW_{i}{}^{2}PW_{j}\\ \;+a_{6}PW_{i}PW_{j}{}^{2}+a_{5}PW_{i}{}^{2}+a_{4}PW_{j}{}^{2}\\ \;+a_{3}PW_{i}PW_{j}+a_{2}PW_{i}+a_{1}PW_{j}+a_{0} \end{array}$$

is the most appropriate model of the overlap in stimulation between two contacts (R^2^ = 0.92 ± 0.11) with the highest average AICc ranking.

### Twitch/tetanic relationship

**Figure 3:**
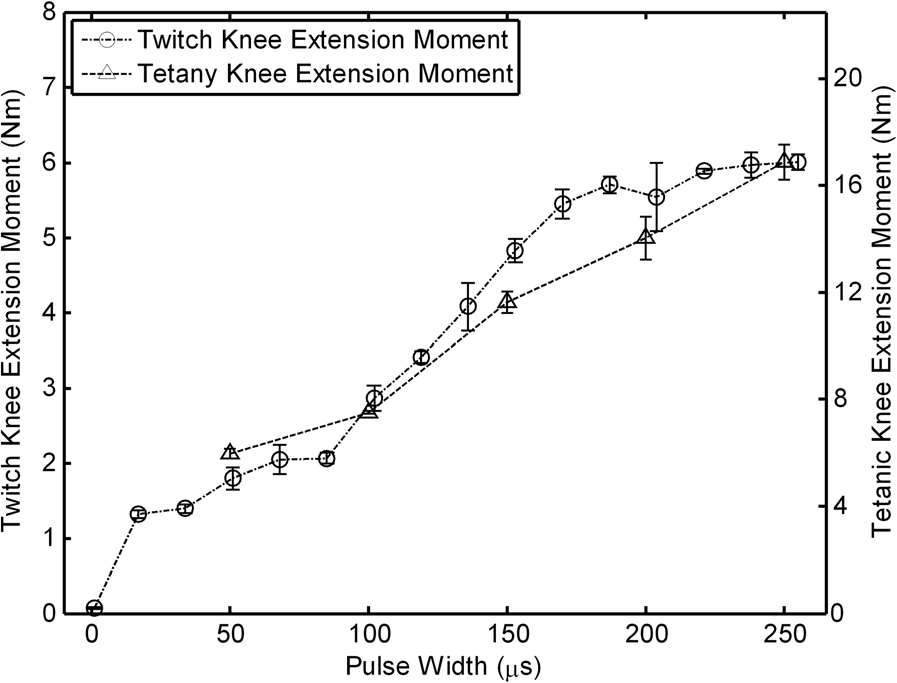
**Twitch/tetanic relationship.** An example of the relationship between twitch (circles) and tetanic (triangles) recruitment curves. A linear scaling factor is calculated as the ratio of the maxima of these curves.

Examples of typical twitch and tetanic recruitment curves are shown in Figure 3. For all sixteen contacts within the four nerve-cuff electrodes, the shape of the twitch recruitment curve was similar to the tetanic curve, and a linear scaling factor was calculated as the ratio of the maxima of the two curves (ratio = 2.56 ± 0.55).

### Optimization of selective stimulation parameters

**Figure 4:**
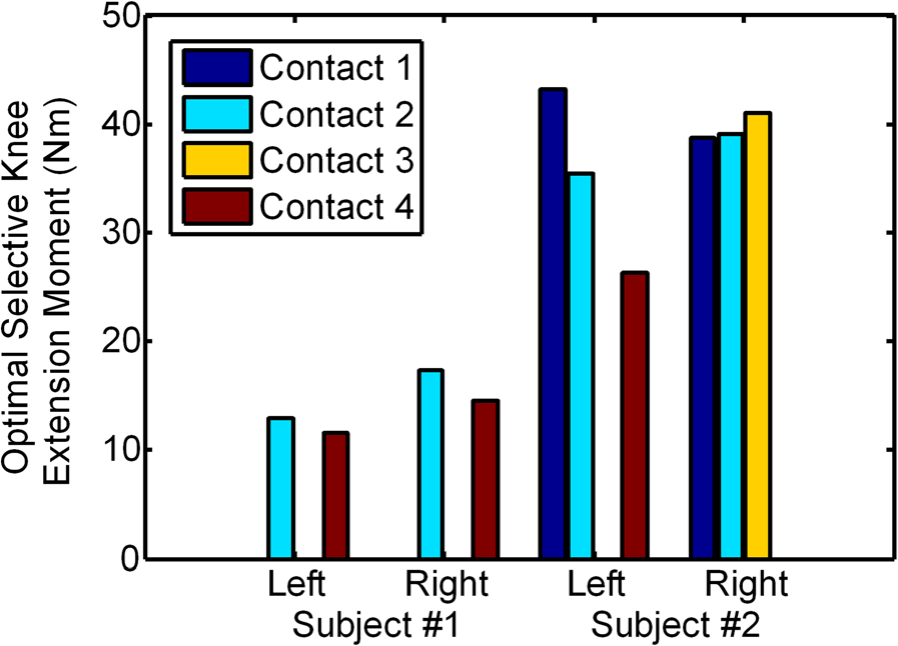
**Optimal selective knee extension moment.** Knee extension moments as a result of optimal selective stimulation parameters selected by minimizing the cost function described above. Every pairwise combination of contacts in each electrode had less than 10% overlap.

Shown in Figure 4 are the joint moments produced by stimulating through each nerve-cuff contact using the optimal stimulation parameters for all nerve-cuff electrodes, as determined by a direct search of the previously defined cost function. Every pairwise combination of contacts within each electrode had less than 10% overlap.

Note that, in Subject 1, only two of four contacts, and in Subject 2, only three of four contacts for either electrode have non-zero stimulus parameters. The results of the optimization consistently demonstrated that removing these one or two contacts from the cost function produced significantly higher joint moments with less overlap than if all contacts were included or other contacts were removed. For contact 3 of the left nerve-cuff electrode in Subject 2, there was never any measurable motor response to stimulation, indicating that it was probably located over sensory axons or connective tissue rather than fascicles containing motor axons.

### Stability of overlap

**Figure 5:**
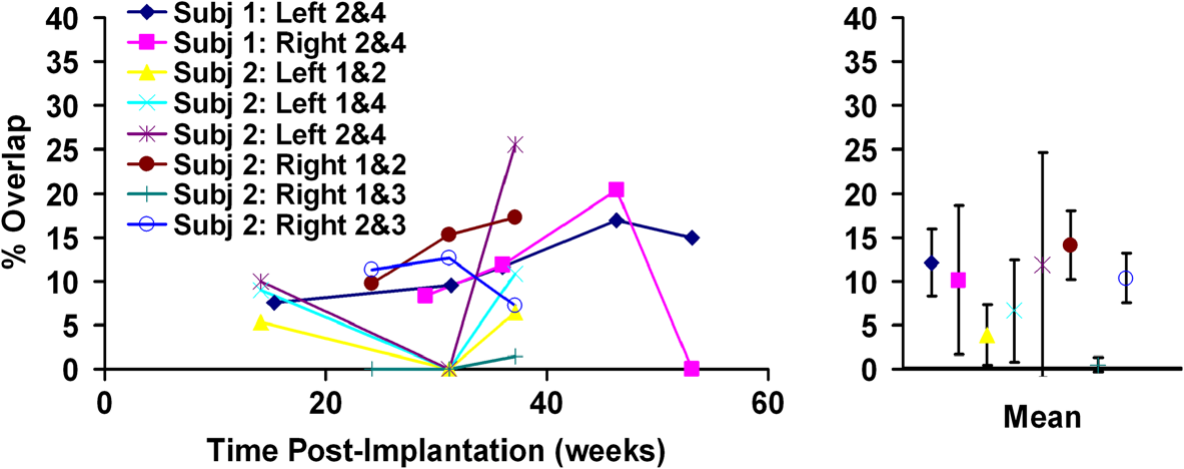
**Stability of overlap in stimulation.** Overlap in stimulation between pairs of contacts in four nerve-cuff electrodes implanted in two human subjects. Also shown to the right are means and standard deviations of overlap for each pair of contacts. No mean was statistically greater than 10%.

Shown in Figure 5 are measurements of overlap in stimulation for contacts within the four nerve-cuff electrodes at multiple time points after implantation along with the mean ± standard deviation for those measurements. Overlap was measured using the optimal selective stimulus parameters quantified at the first time point, so only contacts that produced optimal selectivity at that time were included in subsequent measurements. Therefore, only overlaps for two and three contacts are shown for subjects 1 and 2, respectively. Overlap between most pairs of contacts remained low and constant over time, with only three pairs demonstrating standard deviations greater than 5%. A Student's *t*-test did not demonstrate that mean overlap was statistically greater than 10% for any contact.

## Discussion

The methods presented in this study are designed to optimize stimulation parameters for multi-contact peripheral stimulating electrodes. Results presented here demonstrate that this method can select a set of stimulation parameters that provide strong muscle contractions with low overlap. One of many applications of this technique is in neuroprostheses for standing after paralysis. In the case of these four nerve-cuff electrodes examined in this study, the optimization determined that it is possible for two or three contacts to produce at least 11.6 Nm, but possibly as much as 43.2 Nm of knee extension moment with less than 10% overlap between pairs of contacts. Biomechanical studies estimate that as much as 27% body weight (BW) of knee extension moment is required to keep the knees locked during standing [25]. The sum of selective moments for any one of the nerve-cuff electrodes in this study is greater than these requirements. It is also possible to tailor the results of the optimization by adjusting weighting factors in the cost function to favor larger joint moments at the cost of tolerating more overlap, if necessary. The technique can be applied to any number of real-world situations utilizing multi-contact or multiple single-contact electrodes activating synergistic muscle fiber populations, such as diaphragmatic pacing, hand grasping, or stepping. The methodology can readily be adapted to any electrode technology including high density penetrating arrays or multiple muscle-based or even surface electrodes.

While the optimization method described here was applied to an electrode with only four contacts, it was designed to be scalable to much higher density electrodes. By focusing only on overlap between adjacent pairs of electrodes, and by fitting mathematical models to both overlap and recruitment, it should be possible to select optimal stimulation parameters for electrodes with many more contacts without becoming prohibitively data intensive. If non-adjacent pairwise combinations of contacts were ignored and a limited data set was used to fit the Gompertz and third-order polynomial models, which have three and ten parameters, respectively, the method described above would require approximately 13 hours to completely optimize selective stimulation for a 100-contact Utah slant electrode array, as compared to 11.6 days otherwise [3]. This time requirement is a worst case scenario that only applies if every contact within the electrode activates an agonist population of motor units. In reality, it is likely that many contacts could be removed from the optimization because they activate either sensory neurons or non-agonist populations of motor units. Furthermore, the method used here to identify the relationship between twitch and tetanic recruitment was chosen because of its simplicity, but not its efficiency. In fact, of the estimated 13 hours required to optimize a 100-contact Utah array, nearly 70% of that time would be devoted to characterizing the relationship between twitch and tetanic responses. Other methods described by Durfee, et al. [23], require significantly less time to characterize the relationship between twitch and tetanic responses, and could reduce the worst-case scenario time to completely optimize a 100-contact electrode to approximately six hours.

As presented, the optimization method relies on pulse width modulation, but this is largely a result of the limitations of the implanted stimulator used in this study. While it may be necessary to rely on different mathematical models of recruitment and overlap data, the method should be applicable to pulse amplitude modulation or even modulation of both pulse width and amplitude.

It should be noted that in the case of all four nerve-cuff electrodes presented here, the optimization produced better results if either one or two contacts were eliminated from the optimization. In the case of Subject 1, the two contacts used in the optimization sit opposite one another around the circumference of the nerve, so it is reasonable to expect that they would have less overlap with one another than contacts that are directly adjacent. In the case of Subject 2, one of the contacts that was excluded never demonstrated any motor response to stimulation, which suggests the contact may have been located near sensory neurons or connective tissue.

The results of the optimization were stable over time, with the amount of overlap between the included pairs of contacts remaining largely constant. This suggests that the nerve-cuff electrode provides a stable interface with the nerve, and that only occasional retuning of stimulation parameters would be required to account for changes in muscle strength during clinical implementation of selective stimulation.

The results of this study demonstrate that it is possible to efficiently determine optimal selective stimulation parameters for multi-contact electrodes. Based on these results, it may be possible to use the selectivity achieved here to produce finer control of motor output by recruiting motor units from a small set of contacts for low-force tasks and recruiting from additional contacts for high-force tasks. Additionally, by alternating stimulation between multiple independent populations, it may be possible to reduce the duty cycle of stimulation while maintaining a constant joint moment, and thereby delay the onset of fatigue.

## Conclusions

This study presents a new method for optimizing stimulation parameters for multi-contact peripheral stimulating electrodes. By collecting twitch responses to stimulation and fitting mathematical models to recruitment and overlap data, the method reduces the data requirements for characterizing and optimizing selective stimulation. The use of a cost function that includes terms representing both recruitment and pairwise overlap for all contacts within the electrode allows for maximization of the moments generated by all contacts while simultaneously minimizing the overlaps between all pairs of contacts. This method allows for an objective and automated means of selecting stimulation parameters for electrodes with high densities of contacts, where manual selection of stimulation parameters would be prohibitively time intensive.

The results of this study also suggest that it is possible to generate strong contractions with little or no overlap between contacts within a four-contact spiral nerve-cuff electrode. Overlap in stimulation was also found to be stable over months after implantation, with little variation in overlap in response to the same stimulus parameters applied at multiple time points after implantation.

## Abbreviations

AICc: corrected Akaike information criterion; CWRU: Case Western Reserve University; FNS: functional neuromuscular stimulation; GOF: goodness-of-fit; SCI: spinal cord injury.

## Competing interests

The authors declare that they have no competing interests.

## Authors’ contributions

LEF participated in designing the method, performing experiments, and drafting the manuscript. DJT participated in designing the method and revising the manuscript. RJT participated in designing the method, supervising experiments, and drafting the manuscript. All authors read and approved the final manuscript.
